# miR-629-3p may serve as a novel biomarker and potential therapeutic target for lung metastases of triple-negative breast cancer

**DOI:** 10.1186/s13058-017-0865-y

**Published:** 2017-06-19

**Authors:** Jin Wang, Cailu Song, Hailin Tang, Chao Zhang, Jun Tang, Xing Li, Bo Chen, Xiaoming Xie

**Affiliations:** 1 Department of Breast Oncology, Sun Yat-sen University Cancer Center, State Key Laboratory of Oncology in South China, Collaborative Innovation Center for Cancer Medicine, No.651 Dongfeng East Road, Yuexiu District, Guangzhou, Guangdong 510060 People’s Republic of China; 2 Department of Pathology, Sun Yat-sen University Cancer Center, State Key Laboratory of Oncology in South China, Collaborative Innovation Center of Cancer Medicine, Guangzhou, Guangdong 510060 People’s Republic of China

**Keywords:** miR-629-3p, LIFR, Biomarker, Lung metastasis, Triple-negative breast cancer

## Abstract

**Background:**

Different breast cancer subtypes show distinct tropisms for sites of metastasis. Notably, the lung is the most common site for the first distant recurrence in triple-negative breast cancer (TNBC). The identification of novel biomarkers for lung metastasis is of great importance to improving the outcome of TNBC. In this study, we sought to identify a microRNA (miRNA)-based biomarker and therapeutic target for lung metastasis of TNBC.

**Methods:**

A total of 669 patients without de novo stage IV TNBC were recruited for this study. miRNA profiling was conducted in the discovery cohort. Diagnostic accuracy and prognostic values of candidate miRNAs were evaluated in the training and validation cohorts, respectively. The biological functions of candidate miRNAs, as well as potential targets, were further evaluated through bioinformatic analysis as well as by performing in vitro and in vivo assays.

**Results:**

In the discovery set, we found that miR-629-3p was specifically upregulated in both metastatic foci (fold change 144.16, *P* < 0.0001) and primary tumors (fold change 74.37, *P* = 0.004) in patients with lung metastases. In the training set, the ROC curve showed that miR-629-3p yielded high diagnostic accuracy in discriminating patients with lung metastasis from patients without recurrence (AUC 0.865, 95% CI 0.800–0.930, *P* < 0.0001). Although miR-629-3p predicted poor overall survival and disease-free survival in the validation set, it failed to show significance after multivariate analysis. Notably, logistic regression analyses confirmed that miR-629-3p was an independent risk factor for lung metastasis (OR 4.1, 95% CI 2.5–6.6, *P* < 0.001). Inhibition of miR-629-3p drastically attenuated the viability and migration of TNBC cells, and it markedly suppressed lung metastasis in vivo. Furthermore, we identified the leukemia inhibitory factor receptor (*LIFR*), a well-known metastatic suppressive gene, to be a direct target of miR-629-3p.

**Conclusions:**

miR-629-3p may serve as a novel biomarker and potential therapeutic target for lung metastases of TNBC mediated via LIFR.

**Electronic supplementary material:**

The online version of this article (doi:10.1186/s13058-017-0865-y) contains supplementary material, which is available to authorized users.

## Background

Breast cancer is the most common malignancy in females [1]. It is a heterogeneous disease that is classified into five genetically distinct subtypes [2]. Triple-negative breast cancer (TNBC), characterized by an absence of estrogen receptors (ERs), progesterone receptors (PRs), and human epidermal growth factor receptor 2 (HER2) [3], displays the poorest clinical outcomes, owing to early recurrence and a propensity for distant visceral metastases [4]. Importantly, different breast cancer subtypes show distinct tropisms for sites of metastasis [5, 6]. In particular, the lung is the most common site for first distant recurrence in TNBC, which accounts for 40% of metastatic cases [3]. As such, a better understanding of the molecular mechanisms of lung metastasis and the development of new targeted therapies are of great importance to improving the clinical outcome of TNBC.

So far, there has been minimal research on identifying predictive metastatic biomarkers at specific sites in TNBC [5, 7]. Among many proposed mechanisms underlying metastasis [8, 9], microRNA (miRNA)-regulated transcriptional dynamics has emerged as a critical step [10–13], partially owing to the ability to concurrently target multiple effectors of pathways [14, 15]. miRNA-based anticancer therapies have recently been explored, either alone or in combination with other therapies [16].

In this study, miRNA expression profiles of surgical specimens revealed that miR-629-3p is a specific miRNA associated with lung metastasis in TNBC and is validated as a poor prognostic marker. Although miR-629-3p has been reported to play important roles in cell invasion and metastasis in multiple types of clinically aggressive cancers [17–20], the oncogenic role of miR-629-3p in breast cancer remains unclear. Notably, we identified that the leukemia inhibitory factor receptor (LIFR), which has been proven to be a critical metastasis suppressor [21–26], is a direct target of miR-629-3p.

## Methods

### Clinical specimens and study design

**Fig. 1:**
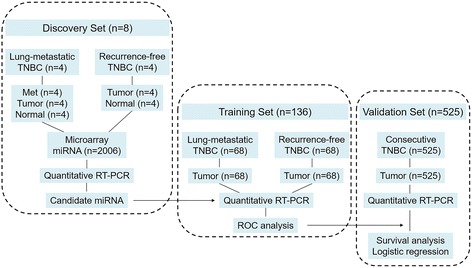
Clinical study design. *Met* Lung metastasis, *Tumor* Primary breast carcinoma, *miRNA* MicroRNA, *Normal* Normal adjacent breast tissue, *RT-PCR* Reverse transcription polymerase chain reaction; *TNBC* Triple-negative breast cancer

Consecutive female patients with infiltrating TNBC who underwent curative surgical treatment (mastectomy or breast-conserving surgery with axillary evaluation) at the Sun Yat-sen University Cancer Center between January 1999 and December 2013 were recruited for this study. Patients with inflammatory breast carcinomas, synchronic bilateral carcinomas, history of other malignant tumors, or incomplete archives of pathological samples were excluded. The subjects’ ER, PR, and HER2 status was evaluated by the department of pathology using immunohistochemistry (IHC) or fluorescence in situ hybridization at the time of diagnosis. TNBC was defined according to the St. Gallen Expert Consensus [27]. Two pathologists independently reevaluated all slides, and any disagreements were resolved by consensus. The pathological tumor stage was assessed according to the criteria described in the seventh edition of the American Joint Committee on Cancer’s *AJCC Cancer Staging Manual*. The tumors were classified into histological grades I–III according to the Nottingham combined histological grading system. Adjuvant chemotherapy and radiation therapy were administered according to the breast cancer guidelines of the National Comprehensive Cancer Network. Follow-up for these patients was completed by 31 December 2015. In brief, after total RNA isolation and quality control, a final cohort of 669 patients with TNBC with formalin-fixed, paraffin-embedded (FFPE) surgical specimens was allocated to either the discovery set, the training set, or the validation set. Figure 1 depicts the different phases of the clinical study design.

#### Discovery set

Four patients with TNBC with lung metastasis underwent pulmonary lobectomy or segmental resection. Paired surgical specimens of lung metastasis (Met), primary breast carcinoma (Tumor), and normal adjacent breast tissue (Normal) were subjected to miRNA profiling. After comparison with Normal, the miRNAs with altered expression levels in both Met and Tumor were deemed to be associated with lung metastasis. Furthermore, primary tumors and corresponding normal breast tissue from another four patients with TNBC with 10-year disease-free survival (DFS) were also prepared for miRNA profiling; the differentially expressed miRNAs in this group were not considered to be involved in promoting lung metastasis.

#### Training set

Primary tumors collected from a larger cohort of patients with lung metastasis (*n* = 68) and patients without recurrence (*n* = 68) were subjected to quantitative reverse transcription polymerase chain reaction (qRT-PCR) analysis of the candidate miRNA. The efficacy of miRNA in diagnosing lung metastasis was determined using the ROC curve.

### Validation set

The prognostic effects of the candidate miRNA for overall survival (OS), DFS, distant metastasis-free survival (DMFS), and locoregional recurrence-free survival (LRRFS) were evaluated in the validation cohort, which was composed of 525 TNBC samples. Correlations between the candidate miRNA and clinicopathologic characteristics were also assessed. Furthermore, associations between the candidate miRNA and sites of distant relapse were evaluated using univariate and multivariate logistic regression models.

### miRNA profiling

miRNA expression profiling was conducted using the Human miRNA V19.0 Microarray (Platform GPL19730, G4872A; Agilent Technologies, Santa Clara, CA, USA), which consists of probes for 2006 human miRNAs based on Sanger miRBase release 19.0. Total RNA was extracted and purified from FFPE tissue using RecoverAll^TM^ Total Nucleic Acid Isolation Kit (Ambion, Austin, TX, USA) according to the manufacturer’s instructions. Microarray image information was converted to spot intensity values using Feature Extraction version 10.7 software (Agilent Technologies). Raw data were normalized by quantile algorithm using GeneSpring software version 12.6 (Agilent Technologies). Logarithmic transformation, using log base 2, was then analyzed. A paired-sample *t* test was used to identify miRNAs with significantly altered expression (fold change >1.5, *P* < 0.05). All microarray data were deposited in the National Center for Biotechnology Information Gene Expression Omnibus (GEO) [GEO:GSE80038]. Venny 2.1.0 (http://bioinfogp.cnb.csic.es/tools/venny/) was used to filter the specific lung metastasis-associated miRNAs for further analysis.

### Quantitative real-time polymerase chain reaction analysis

Total RNA of tissue samples or cells was extracted with TRIzol reagent (Life Technologies, Carlsbad, CA, USA). Reverse transcription of miRNA was done using the TaqMan MicroRNA Reverse Transcription Kit (Applied Biosystems, Foster City, CA, USA). qPCR reactions were performed in triplicate using TaqMan Universal PCR Master Mix (Applied Biosystems) according to the manufacturer’s instructions. Results were quantified using either a 7900 HT sequence detection system (Applied Biosystems) or the IQ^TM^5 Multicolor Real-Time PCR Detection System (Bio-Rad Laboratories, Hercules, CA, USA). All primers were synthesized by Invitrogen (Carlsbad, CA, USA). The expression of miRNA was normalized to U6 small nuclear RNA (snRNA), and fold change was calculated using the comparative cycle threshold (2^−ΔΔ*C*^_T_) method [28].

### Prediction of target genes and enrichment analysis

Putative target genes of miRNAs were predicted using the miRWalk 2.0 database (http://zmf.umm.uni-heidelberg.de/apps/zmf/mirwalk2/miRretsys-self.html) [29], which integrates eight prediction programs, including DIANA-microT, miRanda, miRDB, miRWalk, RNAhybrid, PICTAR2, RNA22, and TargetScan. To increase the accuracy of the prediction, only target genes predicted by at least five programs were retained for further analysis. To annotate the biological functions of candidate miRNAs, the list of target genes was submitted to DAVID Bioinformatics Resources 6.8 (https://david.ncifcrf.gov/tools.jsp), whereby Gene Ontology (GO) function and Kyoto Encyclopedia of Genes and Genomes (KEGG) pathway enrichment analysis were conducted [30]. Pathways with fold enrichment >1.5 and *P* < 0.05 were considered to be of interest.

### Cell lines and animals

Nine human breast cancer cell lines (MDA-MB-453, MDA-MB-468, MDA-MB-231, BT-549, MCF-7, T47D, BT-474, SKBR-3, and HCC-38) and one normal mammary epithelial cell line (MCF-10A) were obtained from the American Type Culture Collection (Manassas, VA, USA). These cells were cultured according to the supplier’s instructions and passaged within 6 months of purchase. Six-week-old female BALB/c nude mice (Animal Experimental Center, Guangdong Academy of Medical Sciences, Guangzhou, China) were maintained under specific pathogen-free conditions at the Laboratory Animal Center of Sun Yat-sen University.

### Transfection and stably engineered cell lines

Cells cultured in six-well plates were transfected with miRNA mimic, antagomiRNA, RNA, short hairpin RNA, and their corresponding negative controls using Lipofectamine™ 2000 reagent (Invitrogen) according to the manufacturer’s instructions. All miRNA oligonucleotides, RNA-expressing lentiviral plasmids, or RNA-interfering lentiviral plasmids were synthesized by GeneCopoeia (Rockville, MD, USA) and are described in Additional file 1: Figure S1. For later in vivo studies, stable inhibition of miR-629-3p in MDA-MB-231 cells and ectopic expression of miR-629-3p in MCF-7 cells were achieved using the pEZX-AM03 and pEZX-MR03 lentiviral delivery systems, respectively. Scrambled nonsilencing vectors were used as negative controls. The stable selection markers were Hygromycin (for anti-miR-629 vectors) and puromycin (for pre-miR-629 vectors). All the vectors were constructed by GeneCopoeia and are described in Additional file 2: Figure S2.

### Cell proliferation assay

Transfected MDA-MB-231 and MCF-7 cells were seeded at a density of 5 × 10^3^ cells per well into 96-well plates and incubated at 37 °C for 24 h. Cell viability was assessed at 24, 48, 72 and 96 h using a 3-(4,5-dimethylthiazol-2-yl)-2,5-diphenyltetrazolium bromide (MTT) assay [31]. Absorbance values were determined at 570 nm (SpectraMax 250 spectrophotometer; Molecular Devices, Sunnyvale, CA, USA).

### Cell migration and invasion assays

Cell migration was examined using wound-healing assays. At 24 h after cell seeding, confluent monolayers were wounded linearly by scraping with sterile 1-ml pipette tips. Progression of migration was observed and photographed using an inverted microscope at 48 h after wounding. The extent of wound closure was assessed using cellSens Dimension software (Olympus Life Science, Waltham, MA, USA).

Cell invasion was analyzed using transwell chamber assays as described previously [31]. Briefly, cells were seeded onto the basement membrane matrix present in the insert of a 24-well extracellular matrix (ECM) culture plate (BD Biosciences, San Jose, CA, USA). Medium containing 10% FBS was added to the lower chamber as a chemoattractant. The noninvading cells and ECM were gently removed with a cotton swab after an additional 48 h. Invasive cells located on the lower side of the chamber were stained with crystal violet, imaged, and counted in five randomly chosen fields.

### Luciferase assays

To determine whether miR-629-3p regulates *LIFR* directly through the interaction with its predicted 3′-untranslated region (UTR) binding sites, luciferase reporter assays were performed using MDA-MB-231 cells. The full length of the *LIFR* 3′-UTR was synthesized by GeneCopoeia and cloned into the pMIR-REPORT luciferase vector (Ambion) using PCR-generated fragments, which served as the wild-type *LIFR* 3′-UTR luciferase vector (wt-LIFR). The first seven complementary nucleotides of *LIFR* bound to the seed region of miR-629-3p were mutated by site-directed mutagenesis (Stratagene, San Diego, CA, USA) to generate the mutant *LIFR* 3′-UTR luciferase vector (mut-LIFR). The wt-LIFR and mut-LIFR were cotransfected with miR-629-3p mimics or scrambled oligonucleotides into MDA-MB-231 cells. Luciferase activity was measured in cell lysates 48 h after transfection using a dual-light luminescent reporter gene assay kit (Applied Biosystems) according to the manufacturer’s instructions.

### Western blotting

Western blotting was performed as described previously [31]. Briefly, proteins extracted from cell lines were transferred to polyvinylidene difluoride membranes (EMD Millipore, Billerica, MA, USA), which were blocked with 5% skim milk powder and incubated with primary anti-LIFR antibody (1:200 dilution, ab101228; Abcam, Cambridge, UK). A peroxidase-conjugated secondary antibody (1:2000 dilution) and enhanced chemiluminescence Western blot detection reagents were used to visualize the target proteins (New England BioLabs, Ipswich, MA, USA), which were quantified with a Bio Image Intelligent Quantifier 1-D (version 2.2.1; Nihon Bio Image Ltd., Tokyo, Japan). An anti-β-actin antibody (Boster Biological Technology, Pleasanton, CA, USA) was used as a protein-loading control.

### Animal studies

For tumor growth analysis, the previously mentioned stably transfected cells (5 × 10^6^ cells/mouse) were orthotopically injected into the mammary fat pads of mice (*n* = 5 in each group). Estradiol pellets (0.72 mg, 60-day release; Innovative Research of America, Sarasota, FL, USA) were implanted subcutaneously into mice with MCF7 cells to induce tumor growth according to previous studies [32]. The size of the tumors was measured every 4 days and calculated following the ellipsoid volume formula: π/6 × length × width^2^ (mm^3^). Primary tumors were excised with the mice under anesthesia 4 weeks after inoculation.

For pulmonary metastasis studies, cells (1 × 10^5^ cells/mouse) were resuspended in 0.1 ml of PBS and injected into the lateral tail veins of mice (*n* = 5 in each group). Progression of lung metastasis was monitored using the Xenogen IVIS Spectrum Imaging System (PerkinElmer, Waltham, MA, USA). After a further 8 weeks, lungs were isolated with the mice under anesthesia, and the number of macroscopically visible pulmonary metastases nodules per mouse was counted by three professional pathology experts, with subsequent validation by hematoxylin and eosin (H&E) staining under a microscope [22].

For histological analysis, slides were stained with H&E for microscopic observations. IHC was performed using the standard streptavidin/peroxidase staining method as previously described [33]. Briefly, after deparaffinization and rehydration, slides were incubated with primary antibody against LIFR (1:50 dilution, ab101228; Abcam) at 4 °C overnight. The immunoreactivity assay was performed using the EnVision^TM^ Detection System (K500711; Dako, Glostrup, Denmark). Results were assessed and captured using an Eclipse 80i microscope (Nikon Instruments, Otawara, Japan).

### Statistical analysis

All statistical analyses were performed with the IBM SPSS Statistics version 22.0 statistical software package (IBM, Armonk, NY, USA). Continuous variables (mean ± SD) and dichotomous variables (frequency and percent) were compared using one-way analysis of variance and the chi-square test, respectively. ROC analysis was conducted to test the specificity and sensitivity of miR-629-3p in predicting lung metastasis. The optimal threshold of miR-629-3p was determined by Youden’s index (by maximizing the sum of sensitivity and specificity) [34], which was used to dichotomize miR-629-3p expression to high or low levels in the validation cohort. Correlations between miR-629-3p and clinicopathologic characteristics were evaluated using Spearman’s rank correlation coefficient presented with *r* values. Survival analysis was conducted using the Kaplan-Meier method with the log-rank test. Significant prognostic factors in the univariate analysis were included in the Cox proportional hazards regression model for multivariate analyses. HRs from the final models were presented with 95% CIs. Risk factors for sites of distant relapse were assessed in multivariate models using logistic regression presented with ORs and 95% CIs. *P* < 0.05 was considered statistically significant.

## Results

### miRNAs associated with lung metastasis

**Fig. 2:**
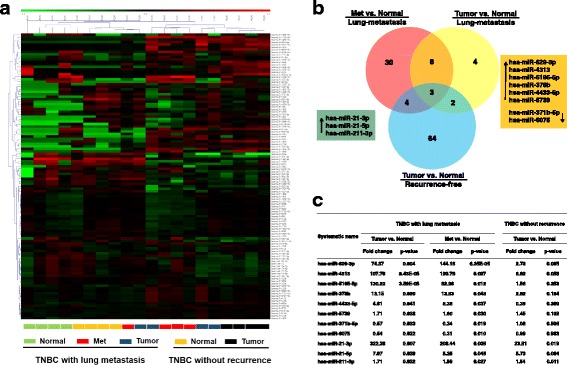
Specific microRNAs (miRNAs) altered in patients with triple-negative breast cancer (TNBC) with lung metastases. **a** Hierarchical clustering represented in the heat map clearly discriminated normal breast tissues from tumors. Samples are presented in columns, and differently expressed miRNAs are presented in rows. *Colored bars* show the range of normalized log_2_ signals. **b** and **c** Venn diagram and the corresponding table describing the expression patterns of miRNAs dysregulated in patients with lung metastases and recurrence-free patients. Three miRNAs were overexpressed regardless of prognosis. Eight miRNAs were dysregulated in both metastatic (Met) and primary tumors (Tumor) in the lung metastasis group, especially miR-629-3p, which was the most upregulated (*P* < 0.001)

All samples in the discovery phase met the quality control parameters recommended by the manufacturer. As shown in Fig. 2a, supervised hierarchical clustering of miRNAs clearly discriminated normal breast tissues from tumors. In addition, the miRNAs (Tumor and Met) in the lung metastasis group clustered separately from miRNAs (Tumor) in the recurrence-free group. Compared with corresponding normal breast tissues, expression of 45 miRNAs and 17 miRNAs in the lung metastasis group was significantly altered in Met and Tumor, respectively, of which 11 miRNAs overlapped (Fig. 2b, c). To distinguish the specific miRNAs associated with lung metastasis, three miRNAs (hsa-miR-21-3p, hsa-miR-21-5p, and hsa-miR-211-3p) were excluded because they were found to be upregulated in primary tumors in the recurrence-free group. The remaining eight dysregulated miRNAs (Fig. 2b) were identified to be putatively involved in the process of lung metastasis, especially miR-629-3p, which was upregulated in both Met and Tumor by more than 70-fold (*P* < 0.001) (Fig. 2c). Subsequent qRT-PCR to reevaluate the expression levels of the eight miRNAs in the discovery set was consistent with microarray results.

### Diagnostic efficacy of miR-629-3p in lung metastasis of TNBC

**Fig. 3:**
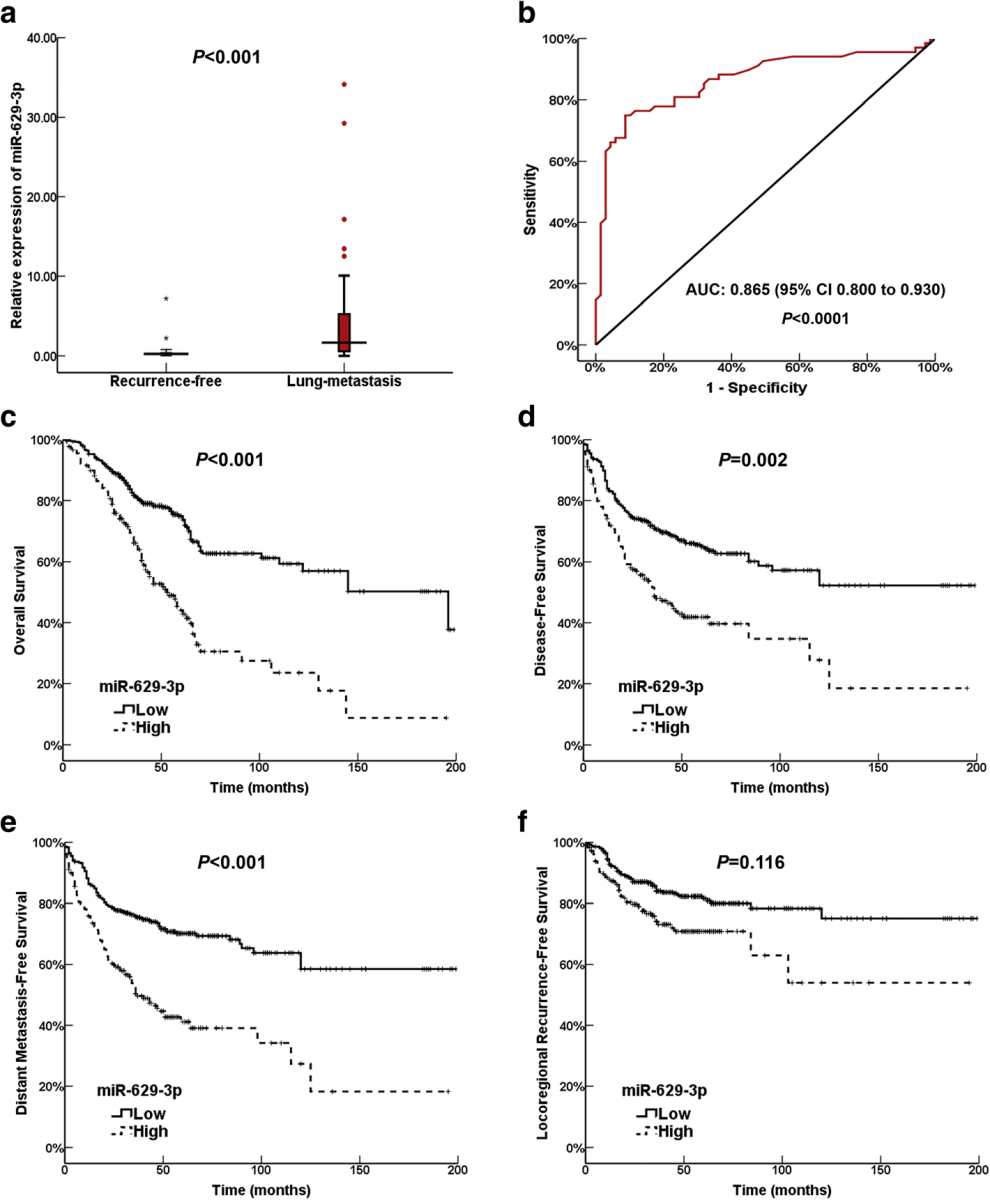
The diagnostic accuracy and prognostic effect of miR-629-3p in patients with triple-negative breast cancer (TNBC) with lung metastasis. **a** In the training set, quantitative real-time polymerase chain reaction analysis of primary tumors indicated that the expression level of miR-629-3p in the lung metastasis group was significantly higher than in the recurrence-free group (*P* < 0.001). **b** ROC curve analysis demonstrated that miR-629-3p was a significant biomarker for diagnosis of lung metastasis, with an AUC of 0.865 (95% CI 0.800–0.930, *P* < 0.0001). **c**, **d**, and **e** High expression of miR-629-3p was correlated with a reduced overall survival (*P* < 0.001), disease-free survival (*P* = 0.002), and distant metastasis-free survival (*P* < 0.001) in univariate analysis. However, miR-629-3p failed to maintain a significant association after adjustment for confounding factors in multivariate analysis. **f** There was no difference in locoregional recurrence-free survival with respect to miR-629-3p expression (*P* = 0.116). *miR* MicroRNA

To further evaluate the findings of the discovery set, the expression levels of miR-629-3p in primary tumors were examined in two independent TNBC cohorts. Characteristics of patients in the training and validation sets are summarized in Additional file 3: Table S1, which were well matched. As shown in Fig. 3a, the expression of miR-629-3p in the lung metastasis group was significantly higher than that in the recurrence-free group (*P* < 0.001). ROC curve analysis demonstrated that miR-629-3p yielded high diagnostic accuracy in discriminating patients with lung metastasis and patients without recurrence (AUC 0.865, 95% CI 0.800–0.930, *P* < 0.0001) (Fig. 3b). When the sensitivity and specificity of miR-629-3p were 75% and 91.3% respectively (maximum of Youden’s index), the corresponding relative expression value of miR-629-3p was 0.6 (normalized against U6 snRNA expression), which was regarded as the optimal cutoff value for further analysis.

### Relationships between miR-629-3p and clinicopathologic parameters

Overexpression of miR-629-3p was observed in 30.1% of the tumors in the validation set. As summarized in Table 1, the Spearman’s correlation coefficients indicated that TNBC cases exhibiting high expression of miR-629-3p were more likely to develop lymphatic metastasis (*r* = 0.139, *P* = 0.001) as well as advanced stage (*r* = 0.135, *P* = 0.002). Notably, a strong positive association between the expression of miR-629-3p and lymphovascular invasion (LVI) was identified (*r* = 0.241, *P* < 0.0001).

**Table 1:** Relationships between miR-629-3p and clinicopathologic factors

Variables	miR-629-3p				Correlation coefficient	*P* value
	Low		High			
	Number of patients (*n* = 367)	%	Number of patients (*n* = 158)	%		
Age, years					−0.016	0.717
< 35	48	13.1	24	15.2		
35–65	291	79.3	121	76.6		
> 65	28	7.6	13	8.2		
Menopause					0.077	0.077
No	230	62.7	86	54.4		
Yes	137	37.3	72	45.6		
T					0.071	0.106
T1	138	37.6	49	31.0		
T2	180	49.0	82	51.9		
T3	32	8.6	17	10.8		
T4	17	4.6	10	6.3		
N					0.139	0.001
N0	184	50.1	54	34.1		
N1	94	25.6	52	32.9		
N2	44	12.0	26	16.5		
N3	45	12.3	26	16.5		
TNM stage					0.135	0.002
I	97	26.4	26	16.5		
II	165	45.0	68	43.0		
III	105	28.6	64	40.5		
Grade					0.054	0.218
I	67	18.3	18	11.4		
II	141	38.4	67	42.4		
III	159	43.3	73	46.2		
Ki-67					0.045	0.298
≤ 14%	102	27.8	37	23.4		
> 14%	265	72.2	121	76.6		
LVI					0.241	<0.001
Negative	326	88.8	109	69.0		
Positive	41	11.2	49	31.0		

*LVI* Lymphovascular invasion

### Prognostic effects of miR-629-3p on TNBC

The prognostic effects of miR-629-3p were evaluated in the validation set. With a median follow-up period of 43 months (range 3–199 months), the 5-year OS and DFS of this cohort were 65.6% and 57.9%, respectively. Univariate and multivariate analyses of OS, DFS, DMFS, and LRRFS are summarized in Table 2. Clinicopathologic variables, including lymphatic metastasis, advanced stage, high grade, and LVI, were established as independent poor prognostic factors for OS, DFS, and DMFS. On one hand, it is noteworthy that high expression of miR-629-3p was associated with a decrease in OS (*P* < 0.001), DFS (*P* = 0.002), and DMFS (*P* < 0.001) in univariate analysis (Fig. 3c–e). On the other hand, there was no difference in LRRFS with respect to miR-629-3p expression (*P* = 0.116) (Fig. 3f). Multivariate analysis demonstrated that miR-629-3p failed to maintain a significant association after adjusting for confounding factors. Regarding the strong association of miR-629-3p with lung metastasis, correlations between miR-629-3p and other specific metastatic sites were further explored.

**Table 2:** Univariate and multivariate analyses of prognostic factors for overall survival, disease-free survival, distant metastasis-free survival, and locoregional recurrence-free survival

Variables	OS				DFS				DMFS				LRRFS			
	Univariate	Multivariate			Univariate	Multivariate			Univariate	Multivariate			Univariate	Multivariate		
	*P* value	HR	95% CI	*P* value	*P* value	HR	95% CI	*P* value	*P* value	HR	95% CI	*P* value	*P* value	HR	95% CI	*P* value
Age (years)	0.478				0.384				0.220				0.245			
Menopause	0.594				0.910				0.852				0.994			
T	<0.001	1.2	0.9–1.5	0.069	<0.001	1.2		0.041	<0.001	1.2	0.9–1.5	0.095	<0.001	1.3	0.9–1.7	0.053
N	<0.001	1.3	1.0–1.6	0.034	<0.001	1.4		0.007	<0.001	1.3	1.1–1.7	0.012	<0.001	1.3	0.9–1.8	0.083
TNM stage	<0.001	1.8	1.1–2.9	0.016	<0.001	1.6		0.021	<0.001	1.9	1.2–2.9	0.007	<0.001	1.4	0.8–2.5	0.247
Surgery	0.774				0.985				0.784				0.610			
Chemotherapy	0.167				0.180				0.192				0.057			
Grade	0.016	1.4	1.1–1.8	0.004	0.003	1.4		0.001	0.002	1.5	1.2–1.9	<0.001	0.254			
Ki-67	0.391				0.499				0.523				0.401			
LVI	<0.001	2.0	1.4–2.9	<0.001	<0.001	1.4		0.047	<0.001	1.6	1.1–2.3	0.008	0.045	0.9	0.6–1.7	0.971
miR-629-3p	<0.001	1.3	0.9–1.8	0.089	0.002	1.1		0.558	<0.001	1.2	0.9–1.7	0.191	0.116			

*Abbreviations: DFS* Disease-free survival, *DMFS* Distant metastasis-free survival, *LRRFS* Locoregional recurrence-free survival, *LVI* Lymphovascular invasion

### Correlations between miR-629-3p and sites of distant metastasis

Of the validation cohort, distant metastasis occurred in 192 (36.6%) patients. Univariate and multivariate analyses of metastatic sites are summarized in Table 3. Multivariate analysis revealed that lymphatic metastasis was an independent risk factor for both lung (*P* = 0.046) and liver (*P* = 0.014) metastases. Meanwhile, advanced stage (II/III) independently increased the risk of bone metastasis (*P* = 0.005). Interestingly, positive LVI was associated with a higher prevalence of relapses in the brain (*P* < 0.001). Notably, univariate analysis showed that high miR-629-3p expression not only correlated with lung metastasis (*P* < 0.001) but also increased the risk of brain metastasis (*P* = 0.002). However, multivariate logistic regression analyses demonstrated that miR-629-3p was an independent risk factor for lung metastasis (OR 4.1, 95% CI 2.5–6.6, *P* < 0.001) but not for brain metastasis (OR 1.6, 95% CI 0.8–3.1, *P* = 0.147).

**Table 3:** Logistic regression analysis of risk factors for metastatic sites

Variables	Lung			Brain			Liver			Bone			Others^a^		
	OR	95% CI	*P* value	OR	95% CI	*P* value	OR	95% CI	*P* value	OR	95% CI	*P* value	OR	95% CI	*P* value
Age	–	–	–	–	–	–	–	–	–	–	–	–	–	–	–
Menopause	–	–	–	–	–	–	–	–	–	–	–	–	–	–	–
T3/4	1.1	0.7–1.5	0.748	1.4	0.8–2.2	0.167	1.5	0.9–2.3	0.080	0.9	0.6–1.2	0.435	1.7	1.0–3.0	0.049
N1/2/3	1.5	1.0–2.2	0.046	0.8	0.5–1.3	0.348	1.9	1.1–3.1	0.014	1.2	0.8–1.8	0.330	1.3	0.7–2.3	0.405
TNM II/III	1.7	0.8–3.4	0.143	2.0	0.8–5.2	0.138	1.0	0.4–2.6	0.994	3.0	1.4–6.5	0.005	1.4	0.4–4.7	.597
Surgery	–	–	–	–	–	–	–	–	–	–	–	–	–	–	–
Chemotherapy	–	–	–	–	–	–	–	–	–	–	–	–	–	–	–
Grade II/III	1.3	0.9–1.9	0.105	1.7	1.0–2.8	0.039	–	–	–	1.6	1.1–2.3	0.026	1.9	1.0–3.9	0.044
Ki-67	–	–	–	–	–	–	–	–	–	–	–	–	–	–	–
LVI (positive)	0.9	0.5–1.7	0.863	4.1	1.9–8.4	<0.001	1.1	0.5–2.4	0.760	1.3	0.7–2.4	0.371	–	–	–
miR-629-3p (high)	4.1	2.5–6.6	<0.001	1.6	0.8–3.1	0.147	–	–	–	–	–	–	–	–	–

*LVI* Lymphovascular invasion

A blank (–) field indicates that the variable was not significant in the univariate analysis and was not included in the multivariate analysis

^a^“Others” refers to distant nodal, pleural/peritoneal, abdominal/mediastinal viscera, spinal cord, eye, and other unclassified organs

### GO annotation and KEGG pathway analysis of miR-629-3p

Target genes of miR-629-3p predicted by eight programs are listed in Additional file 4: Table S2. A total of 2267 target genes were predicted by at least five programs. GO annotation analysis (Additional file 5: Table S3) indicated that the biological process of the candidate targets was significantly (fold enrichment >1.5, *P* < 0.05) related to cell proliferation, cell migration, cell-matrix adhesion, blood vessel development, and lymph vessel development. KEGG analysis identified that the set of candidate targets of miR-629-3p was significantly enriched in 52 pathways (fold enrichment >1.5, *P* < 0.05), including several well-known oncogenic signaling pathways such as Ras (*P* = 0.0002), Hippo (*P* = 0.0005), transforming growth factor (TGF)-β (*P* = 0.0009), mitogen-activated protein kinase (MAPK) (*P* = 0.001), phosphoinositide 3-kinase (PI3K)-Akt (*P* = 0.003), and Wnt (*P* = 0.041), as shown in Additional file 6: Table S4.

### miR-629-3p promotes proliferation, migration, and invasion of TNBC in vitro

**Fig. 4:**
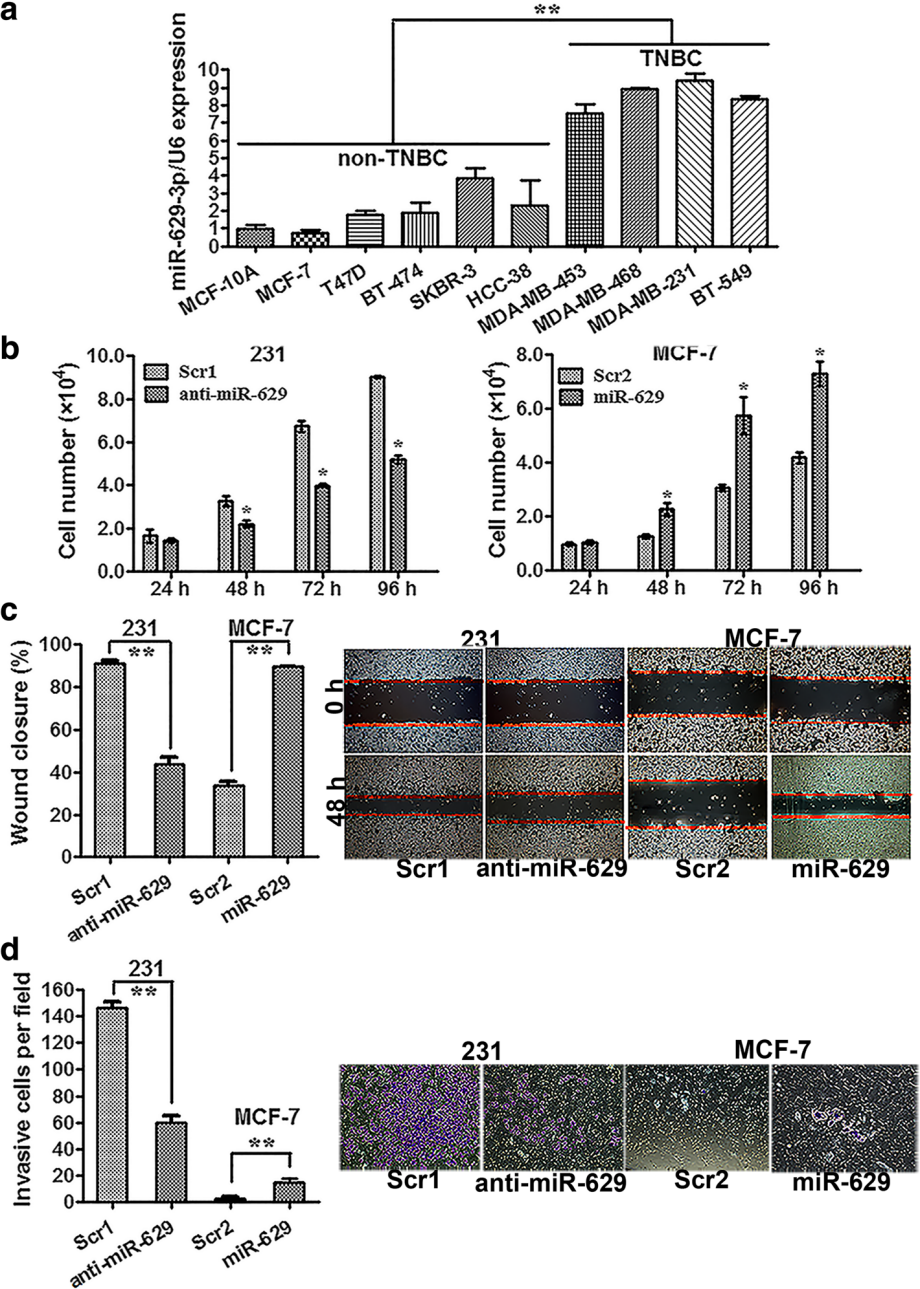
miR-629-3p promotes cell proliferation and invasion of triple-negative breast cancer (TNBC). **a** The relative expression levels of miR-629-3p in four highly metastatic TNBC cell lines were significantly higher than in non-TNBC cell lines. **b** 3-(4,5-Dimethylthiazol-2-yl)-2,5-diphenyltetrazolium bromide assay results showed that the viability of MDA-MB-231 cells was markedly suppressed by an inhibitor of miR-629-3p (anti-miR-629) compared with scrambled oligonucleotide 1 (Scr1). However, ectopic expression of miR-629-3p considerably increased the viability of MCF-7 cells compared with scrambled oligonucleotide 2 (Scr2). **c** and **d** Analysis of cellular migration and invasion using the wound-healing and transwell assays demonstrated that enforced upregulation of miR-629-3p significantly enhanced the migration and invasion capability of MCF-7 cells, whereas inhibition of miR-629-3p dramatically attenuated the migration and invasion capability of MDA-MB-231 cells. Representative images of the assays are shown in the *right panel*. Original magnification × 200. All data are presented as an average of triplicate measurements with SE. *P < 0.05 and ** *P* < 0.01

miR-629-3p expression was detected in nine breast cancer cell lines and one mammary epithelial cell line by qRT-PCR analysis. It is of particular note that miR-629-3p exhibited a pronounced upregulation in four highly metastatic TNBC cell lines (MDA-MB-453, MDA-MB-468, MDA-MB-231, and BT-549) (*P* < 0.01) (Fig. 4a), suggesting that miR-629-3p is likely associated with subtype specificity and the development of metastasis. According to the expression levels of miR-629-3p, MDA-MB-231 cells and MCF-7 cells were used in further assays.

To analyze the biological effects of miR-629-3p on TNBC, MDA-MB-231 cells were transfected with an inhibitor of miR-629-3p (anti-miR-629) or scrambled oligonucleotide 1 (Scr1), and MCF-7 cells were transfected with an miR-629-3p mimic (miR-629) or scrambled oligonucleotide 2 (Scr2). The relative expression levels of miR-629-3p in MDA-MB-231 and MCF-7, which included positive and negative controls for miR-629-3p, are shown in Additional file 7: Figure S3. At 48 h posttransfection, the MTT assay showed that the viability of MDA-MB-231 cells was significantly reduced by anti-miR-629 compared with the Scr1-transfected cells (*P* < 0.05). However, ectopic expression of miR-629-3p considerably improved the viability of MCF-7 cells (*P* < 0.05), as shown in Fig. 4b. Analysis of metastasis-related cell motility, such as cellular migration and invasion, using the wound-healing and transwell assays demonstrated that enforced upregulation of miR-629-3p significantly increased the migration and invasion capability of MCF-7 cells, whereas inhibition of miR-629-3p dramatically attenuated the migration and invasion capability of MDA-MB-231 cells (*P* < 0.01), as shown in Fig. 4c, d.

### miR-629-3p directly targets the 3′-UTR of *LIFR*

According to miRNA binding site enrichment analysis, the putative targets of miR-629-3p are tumor suppressor genes (*P* = 0.013). The TSGene 2.0 database for updated tumor suppressor genes and their features in pan-cancer (https://bioinfo.uth.edu/TSGene/download.cgi) [35] identified *LIFR* as the only tumor suppressor gene that was predicted by all eight programs.

**Fig. 5:**
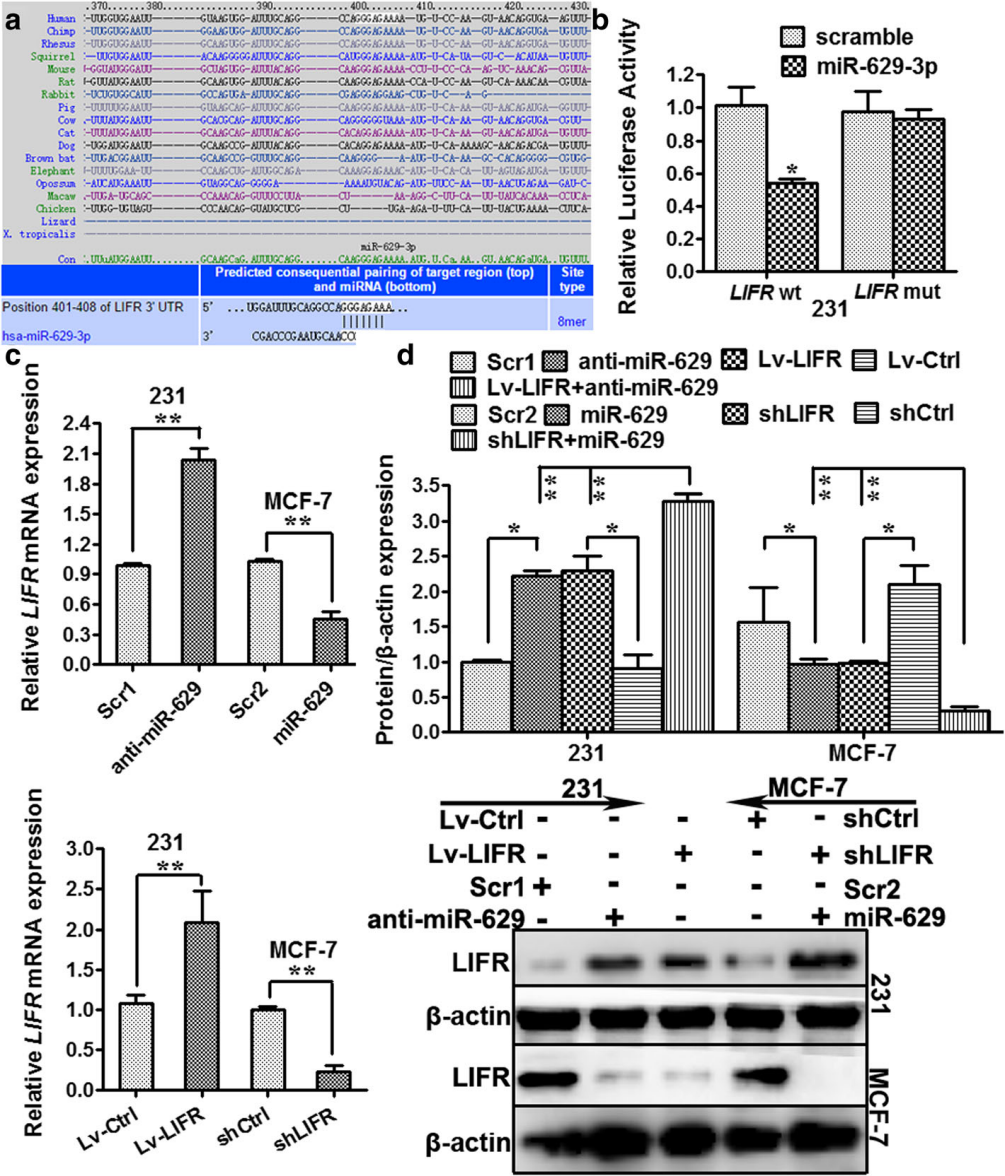
Leukemia inhibitory factor receptor (LIFR) is a direct target of miR-629-3p. **a** The putative sequence of the *LIFR* 3′-untranslated region bound to the seed region of miR-629-3p. **b** The luciferase activity of wild-type *LIFR* reporter constructs in MDA-MB-231 cells exhibited a distinct reduction after cotransfection with miR-629-3p compared with those cotransfected with scrambled oligonucleotides. However, mutant *LIFR* abrogated the posttranscriptional inhibitory effect of miR-629-3p. **c** Quantitative real-time polymerase chain reaction analysis indicated that transfection of MCF-7 cells with miR-629 or *LIFR* short-hairpin RNA interfering plasmid (shLIFR) led to a significant reduction in *LIFR* messenger RNA (mRNA) levels. Meanwhile, transfection with anti-miR-629 or *LIFR*-expressing lentiviral plasmid (Lv-LIFR) increased the expression of *LIFR* in MDA-MB-231 cells compared with the corresponding negative controls. **d** Western blot analysis showed that transfection of MCF-7 cells with miR-629, shLIFR, or shLIFR + miR-629 led to a significant reduction in LIFR protein levels (*P* < 0.05), especially in the shLIFR + miR-629 group (*P* < 0.01). Meanwhile, transfection with anti-miR-629, Lv-LIFR, or Lv-LIFR + anti-miR-629 increased the expression of LIFR in MDA-MB-231 cells significantly (*P* < 0.05), especially in the Lv-LIFR + anti-miR-629 group (*P* < 0.01). The inhibitory effect of miR-629 and shLIFR on LIFR expression was similar and also promoted the effect of anti-miR-629 and Lv-LIFR. * *P* < 0.05 and ** *P* < 0.01

The 3′-UTR of *LIFR*, which contains the miR-629-3p putative binding site, is shown in Fig. 5a. Compared with those cotransfected with scrambled oligonucleotides, the luciferase activity of wt-LIFR reporter constructs in MDA-MB-231 cells exhibited a distinct reduction after cotransfection with miR-629-3p (*P* < 0.05). In contrast, mutations in the 3′-UTR of *LIFR* bound to the seed region of miR-629-3p abrogated the posttranscriptional inhibitory effect of miR-629-3p (*P* > 0.05) (Fig. 5b).

Furthermore, we transfected the miR-629 or *LIFR*-interfering plasmid (shLIFR) into MCF-7 cells, both of which led to a significant reduction in *LIFR* mRNA expression (*P* < 0.01). Meanwhile, transfection with anti-miR-629 or the *LIFR*-expressing plasmid (Lv-LIFR) increased the expression of *LIFR* in MDA-MB-231 cells compared with the corresponding negative controls (*P* < 0.01), as shown in Fig. 5c.

To further confirm the link between LIFR and miR-629-3p, we transfected miR-629, shLIFR, or shLIFR + miR-629 into MCF-7 cells, all of which led to a significant reduction in LIFR protein levels (*P* < 0.05), especially in the shLIFR + miR-629 group (*P* < 0.01). Meanwhile, transfection with anti-miR-629, Lv-LIFR, or Lv-LIFR + anti-miR-629 increased the expression of LIFR in MDA-MB-231 cells significantly (*P* < 0.05), especially in the Lv-LIFR + anti-miR-629 group (*P* < 0.01), as shown in Fig. 5d. The inhibitory effect of miR-629 and shLIFR on LIFR expression was similar and also promoted the effect of anti-miR-629 and Lv-LIFR. The above results validated that LIFR was a bona fide target of miR-629-3p.

### miR-629-3p promotes tumorigenesis and lung metastasis of TNBC in vivo

**Fig. 6:**
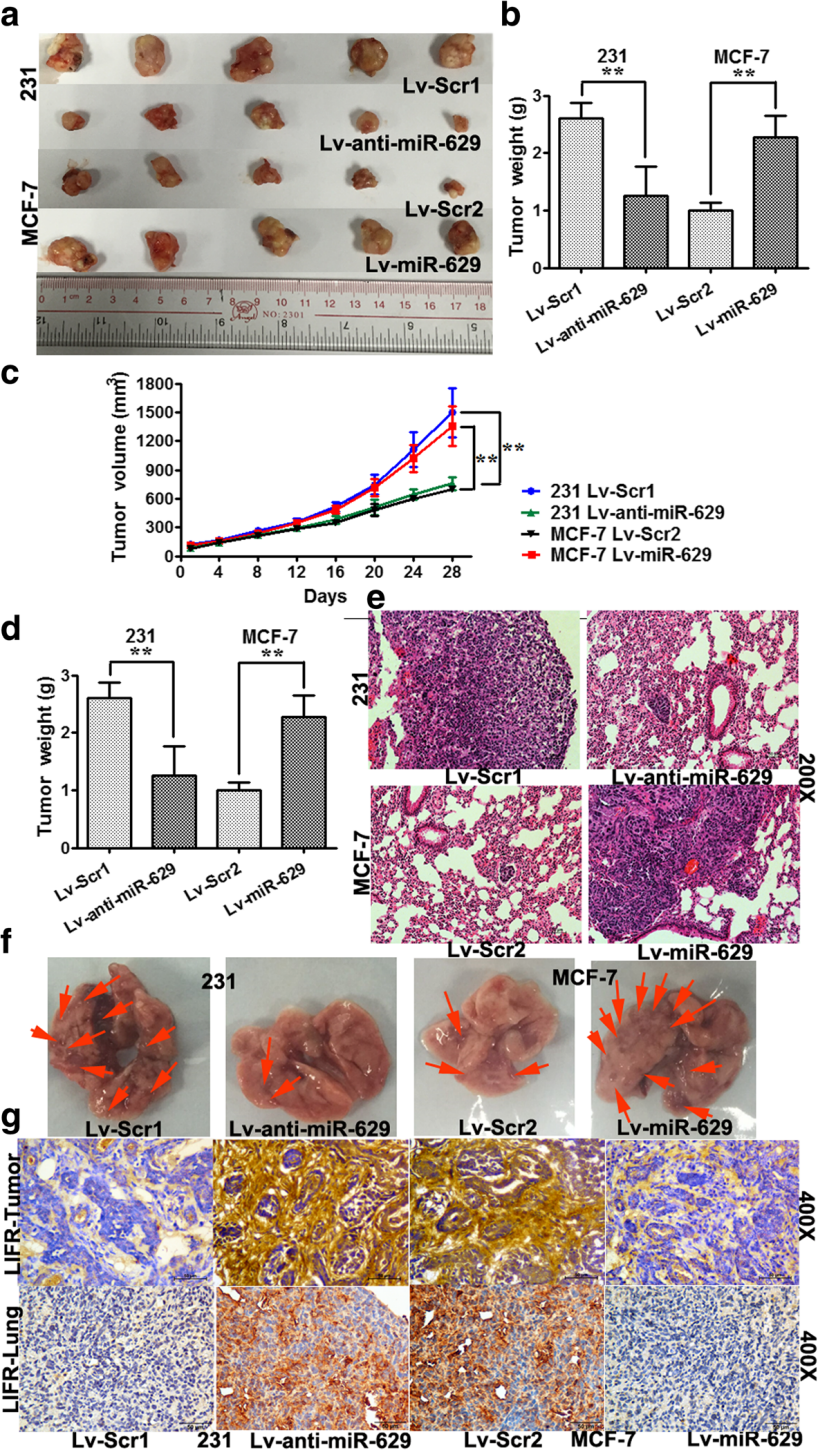
miR-629-3p promotes tumorigenesis and lung metastasis of triple-negative breast cancer (TNBC). **a**, **b**, and **c** Stably engineered miR-629-3p-inhibiting MDA-MB-231 cells (Lv-anti-miR-629) and pre-miR-629-expressing MCF-7 cells (Lv-miR-629) were inoculated into the mammary gland fat pads of nude mice. The weight and volume of mammary tumors generated from Lv-anti-miR-629 were significantly less than those from scrambled 1 MDA-MB-231 cells (Lv-Scr1). Likewise, compared with scrambled 2 MCF-7 cells (Lv-Scr2), Lv-miR-629 cells showed a significant increase in tumor growth. **d** The number of macroscopically visible pulmonary metastatic nodules per mouse was counted. Lv-anti-miR-629 decreased the number of metastatic foci in lungs dramatically in mice with MDA-MB-231 tumors. However, Lv-miR-629 increased the lung metastases in mice with MCF-7 tumors. **e** Lung metastases were confirmed by hematoxylin and eosin staining (original magnification × 200). **f** *Arrows* indicate visible metastatic nodules in representative gross specimens of lung sections. **g** Immunohistochemical analysis further substantiated that miR-629-3p targeted leukemia inhibitory factor receptor (*LIFR*) in both primary tumors and lung metastases (original magnification × 400; *insets*, original magnification × 200). * *P* < 0.05 and ** *P* < 0.01

Stably engineered miR-629-3p-inhibiting MDA-MB-231 cells (Lv-anti-miR-629) and pre-miR-629-expressing MCF-7 cells (Lv-miR-629) were inoculated into the mammary gland fat pads of nude mice. We found that the mammary tumors generated from miR-629-3p-inhibiting MDA-MB-231 cells were significantly smaller than those from scrambled 1-MDA-MB-231 cells (Lv-Scr1) (*P* < 0.01). Likewise, compared with scrambled 2-MCF-7 cells (Lv-Scr2), ectopic expression of miR-629-3p in MCF-7 cells increased tumor growth significantly (*P* < 0.01) (Fig. 6a–c).

To further examine the effects of miR-629-3p on lung metastasis in vivo, we injected the aforementioned MDA-MB-231 and MCF-7 derivatives into the lateral tail veins of 6-week-old female BALB/c mice. As shown in Fig. 6d, mice injected with MDA-MB-231 cells (Lv-Scr1) displayed prominent lung metastases, whereas mice injected with MCF-7 cells (Lv-Scr2) were less likely to develop lung metastases. The number of metastatic foci in lungs was decreased dramatically by suppression of miR-629-3p in mice with MDA-MB-231 tumors (Lv-anti-miR-629) (*P* < 0.01) and was increased by ectopic expression of miR-629-3p in mice with MCF-7 tumors (Lv-miR-629) (*P* < 0.01). The gross specimens and H&E staining of the lung sections are shown in Fig. 6e and f. IHC analysis further substantiated that miR-629-3p targeted LIFR in both primary tumors and metastatic foci of TNBC with lung metastasis (Fig. 6 g).

## Discussion

In patients with breast cancer, lung metastasis gene signatures have been preliminarily identified [7]. Nevertheless, the precise molecular mechanisms for the altered expression of miRNAs in lung metastasis of TNBC are unclear. This is the first report showing that miR-629-3p acts as a specific predictor for lung metastasis in TNBC.

Because the characteristics of a primary tumor are usually preserved in metastases [36], we reasoned that dysregulated expression of key miRNAs that promote a highly invasive phenotype of TNBC would be present in both the primary and metastatic lesions. However, much of the current literature is focused on considering the entire dataset in the process of identifying differentially expressed genes in cancer and ignores individual biological variations [37]. Therefore, we allowed for the heterogeneity of miRNAs in different individuals by using paired samples of primary tumors, metastatic foci, and normal breast tissues from each patient with lung metastasis to evaluate miRNA dysregulation in microarray analyses. miRNA profiling revealed that miR-629-3p is most commonly upregulated in both metastatic lesions and primary carcinomas in patients with TNBC with lung metastasis compared with normal breast tissue. In addition, there was no difference in the expression levels of miR-629-3p between primary tumors and normal breast tissues from patients with TNBC without any recurrence. We then confirmed the predictive capability of miR-629-3p on lung metastasis in two independent TNBC cohorts. Furthermore, in the validation cohort, high expression of miR-629-3p exhibited a strong positive association with lymphatic metastasis and LVI. Notably, we also assessed the relationships between miR-629-3p and other distant organs, including the brain, liver, and bone. Interestingly, miR-629-3p was strongly associated with brain metastasis but failed to retain a significant association after multivariate analysis; this suggests that miR-629-3p may act in an organ-specific manner. Subsequent in vitro and in vivo assays supported our findings that miR-629-3p increased the risk of lung metastasis.

Aberrant elevation in the expression of miR-629-3p has been described in various carcinomas, including liver [17], lung [38, 39], colon, lymphoma, ovary, prostate, and testis [20], suggesting that miR-629-3p fulfills a tumor-promoting role in these contexts. However, little is known about the mechanism of miR-629-3p dysregulation in breast cancer [18, 19]. In the present study, anti-miR-629-3p drastically reduced the proliferative and migratory capability of MDA-MB-231 cells (TNBC and metastatic). Given that miR-629-3p is expressed at considerably higher levels in TNBC cells than in luminal breast cancer cells, we hypothesized that the signaling pathways promoting lung metastasis are partially shared by the two different breast cancer subtypes, in which the miR-629-3p/LIFR axis plays an important role regardless of ER expression, which requires further study. Although miR-629 has been reported to suppress apoptosis through a miRNA-inflammatory feedback loop circuit in hepatocellular carcinoma [17], we found no evidence that apoptosis was affected by miR-629-3p in TNBC cells (data not shown). This discrepancy suggests that the oncogenic roles of miR-629-3p vary in different cancer types and that these roles are mediated through different direct or indirect targets.

With regard to the validated targets of miR-629-3p, Hatziapostolou et al. demonstrated a novel miRNA feedback inflammatory loop in hepatocellular carcinoma involving miR-629, which initiated hepatocellular carcinogenesis by suppressing hepatocyte nuclear factor 4α [17]. miR-629 is also reported to modulate the expression of the *Nbs1* gene, which induces DNA repair and increases lung cancer risk [38]. To identify target genes more precisely, we performed in silico analysis using eight miRNA prediction databases combined with GO enrichment and KEGG pathway analyses. As a result, predicted targets were significantly enriched in many known oncogenic signaling pathways, such as Ras, TGF-β, Hippo, MAPK, 5′-adenosine monophosphate-activated protein kinase, PI3K-Akt, focal adhesion, Wnt, and tumor necrosis factor, providing convincing evidence of the oncogenic role of miR-629-3p in TNBC. Moreover, we focused on several tumor suppressor genes; of these, *LIFR* was a promising candidate target gene of miR-629-3p after evaluation with qRT-PCR, Western blotting, and luciferase reporter assays.

LIFR belongs to the gp130 receptor family, which has been recognized as a tumor suppressor gene in multiple types of cancers [40], including breast [21–23], hepatocellular [24–26], and pancreatic cancers [41]. Remarkably, two recent reports have highlighted LIFR as a novel metastasis suppressor in breast cancer. Johnson et al. demonstrated that loss of LIFR allowed dormant breast cancer cells to proliferate and specifically colonize in the bone. Additionally, they found that breast cancer cells which aggressively colonize the lung also lack a functional *LIFR* and do not respond to LIF in vitro [21]. In parallel, Ma et al. found that *LIFR* was inversely correlated with lung metastasis in breast cancers, which was identified as the downstream of miR-9 and upstream of Hippo signaling [22]. Additionally, we also note the study by Nandy et al. [42], who demonstrated that miR-125a influenced stem cells by regulating Hippo signaling through LIFR in human primary breast cancer cells. Interestingly, in the discovery set of our study, we found that miR-125b was statistically downregulated in TNBC with good prognosis (fold change 2.3, *P* = 0.005). These data can be found in the GEO database under accession number [GEO:GSE80038]). Moreover, *LIFR* has been reported to inhibit metastasis by negatively regulating the PI3K-Akt-matrix metalloproteinase 13 cascade in hepatocellular carcinoma [26]. Remarkably, in the present study, KEGG pathway analysis demonstrated that the Hippo signaling pathway (*P* = 0.00049, fold enrichment 1.99) and PI3K-Akt signaling pathway (*P* = 0.00327, fold enrichment 1.50) were both significantly affected by miR-629-3p. Taken together, the interactive miRNAs and associated pathways regulating LIFR are of great potential to be investigated in future studies.

## Conclusions

The identification of a sensitive and specific biomarker predicting the lethal metastasis of TNBC can enable detection of a relapse as early as possible to promote the survival of patients and reduce treatment costs. The present study shows the independent predictive effect of miR-629-3p on lung metastasis in TNBC and reveals that the suppression of miR-629-3p attenuates pulmonary metastasis in experimental breast cancer by directly targeting LIFR, which is an inhibitor of multiple metastatic signaling pathways. Therefore, future studies need to elucidate the specific contributions of miR-629-3p in mediating these predicted pathways, as well as to identify the mechanism of miR-629-3p stimulation in TNBC, which remains crucial to the development of targeted therapies.

## Additional files

Additional file 1: Figure S1. Sequences and structures of miR-629-3p mimics, inhibitor of miR-629-3p, and *LIFR*-expressing and *LIFR*-interfering lentivirus plasmids. (PDF 280 kb)

Additional file 2: Figure S2. Sequences and structures of anti-miR-629-3p lentiviral vectors, pre-miR-629 lentiviral vectors, and their corresponding scrambled vectors. (PDF 631 kb)

Additional file 3: Table S1. Characteristics of patients with TNBC in the training set and validation set. (PDF 142 kb)

Additional file 4: Table S2. Target genes of miR-629-3p predicted by eight programs. (XLSX 2295 kb)

Additional file 5: Table S3. GO annotation analysis of miR-629-3p. (XLSX 24 kb)

Additional file 6: Table S4. KEGG pathway analysis of miR-629-3p. (XLSX 15 kb)

Additional file 7: Figure S3. Transfection of miR-629, anti-miR-629, Scr1, and Scr2 in MDA-MB-231 and MCF-7 cells. (PDF 53 kb)

## Abbreviations

DFS: Disease-free survival
DMFS: Distant metastasis-free survival
ECM: Extracellular matrix
ER: Estrogen receptor
FFPE: Formalin-fixed, paraffin-embedded
GEO: Gene Expression Omnibus
GO: Gene Ontology
H&E: Hematoxylin and eosin
HER2: Human epidermal growth factor receptor 2
IHC: Immunohistochemistry
KEGG: Kyoto Encyclopedia of Genes and Genomes
LIFR: Leukemia inhibitory factor receptor
LRRFS: Locoregional recurrence-free survival
LVI: Lymphovascular invasion
Lv-LIFR: *LIFR*-expressing lentiviral plasmid
MAPK: Mitogen-activated protein kinase
Met: Lung metastasis
miRNA: MicroRNA
Normal: Normal adjacent breast tissue
MTT: 3-(4,5-dimethylthiazol-2-yl)-2,5-diphenyltetrazolium bromide
mut-LIFR: Mutant *LIFR* 3′-untranslated region luciferase vector
OS: Overall survival
PI3K: Phosphoinositide 3-kinase
PR: Progesterone receptor
qRT-PCR: Quantitative real-time polymerase chain reaction
Scr1: Scrambled oligonucleotide 1
Scr2: Scrambled oligonucleotide 2
shLIFR: *LIFR* short-hairpin RNA plasmid
snRNA: Small nuclear RNA
TGF-β: Transforming growth factor-β
TNBC: Triple-negative breast cancer
Tumor: Primary breast carcinoma
UTR: Untranslated region
wt-LIFR: wild-type *LIFR* 3′-untranslated region luciferase vector
