# Supplementary material for: miR-629-3p may serve as a novel biomarker and potential therapeutic target for lung metastases of triple-negative breast cancer
Source: Breast Cancer Res. 2017 Jun 19;19:72. doi: 10.1186/s13058-017-0865-y (PMC5477310; doi:10.1186/s13058-017-0865-y)

miR-629-3p mimics (miR-629)

GeneCopoeia™ Expressway to Discovery
9620 Medical Center Drive, Suite 101
Rockville, MD 20850
Tel: 301-762-0888
Fax: 301-762-3888
Web: www.genecopoeia.com
Email: sales@genecopoeia.com

miRNA Mimic Datasheet

Small RNA duplex for miRNA function study

Product Information

**Catalog No.:**	HmiR-SN0738
**Description:**	A chemically synthesized RNA duplex mimicing endogenous miRNA function
**Sequence:**	5'- GUUCUCCCAACGUAAGCCCAGC -3'<br>3'- UUCAAGAGGGUUGCAUUCGGGU -5'
**Amount:**	6 nmol
**Format:**	Powder
**Purification:**	HPLC
**Quality Assurance:**	The integrity of the RNA duplex is confirmed by PAGE Analysis

miRNA Info

**miRNA Name:**	hsa-miR-629-3p
**miRNA Accession:**	MIMAT0003298
**miRNA Sequence:**	GUUCUCCCAACGUAAGCCCAGC

Please refer to product manual for further details.

GeneCopoeia is committed to providing our customers with high-quality products. If you should have any questions or concerns about any GeneCopoeia products, please contact us at 301-762-0888.

GeneCopoeia Products are for Research Use Only
Copyright © 2010 GeneCopoeia Inc.

inhibitor of miR-629-3p (anti-miR-629)

GeneCopoeia™ Expressway to Discovery
9620 Medical Center Drive, Suite 101
Rockville, MD 20850
Tel: 301-762-0888
Fax: 301-762-3888
Web: www.genecopoeia.com
Email: sales@genecopoeia.com

miArrest™ miRNA Synthetic inhibitors Datasheet

Single stranded and chemically modified for mature miRNA knockdown

Product Information

**Catalog No.:**	HmiR-AN0738-SN-10
**Content:**	A modified single-stranded RNA Oligonucleotide
**Sequence:**	GCUGGGCUUACGUUGGGAGAAC
**Modification:**	2'-O-Methyl-RNA
**Amount:**	10 nmol
**Appearance:**	Powder
**Purification:**	HPLC
**Identity:**	The mass of the single-stranded RNA oligonucleotides is identified using PAGE Analysis

Target miRNA Info

**miRNA Name:**	hsa-miR-629-3p
**miRNA Accession:**	MIMAT0003298
**miRNA Sequence:**	GUUCUCCCAACGUAAGCCCAGC

Please refer to product manual for further details.

GeneCopoeia is committed to providing our customers with high-quality products. If you should have any questions or concerns about any GeneCopoeia products, please contact us at 301-762-0888.

GeneCopoeia Products are for Research Use Only
Trademarks: GeneCopoeia™, miArrest™ (GeneCopoeia Inc).
Copyright © 2012 GeneCopoeia Inc.
MIO-041712

LIFR expressing plasmid (Lv-LIFR)

GeneCopoeia™ Expressway to Discovery www.genecopoeia.com

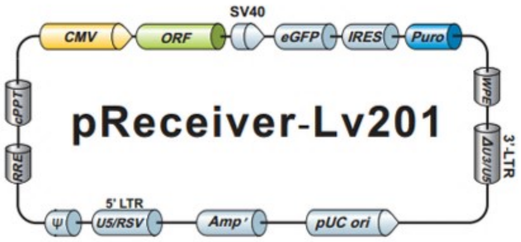

LIFR interfering plasmid (shLIFR)

GeneCopoeia™ Expressway to Discovery www.genecopoeia.com

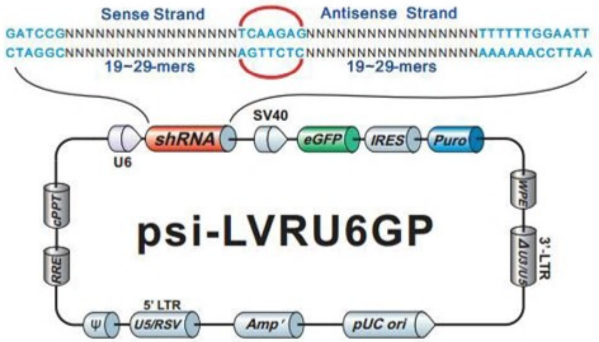

Supplement: Supplementary file 1 — Sequences and structures of miR-629-3p mimics, inhibitor of miR-629-3p, and LIFR-expressing and LIFR-interfering lentivirus plasmids. (PDF 280 kb) [file 13058_2017_865_MOESM1_ESM.pdf]
