# Supplementary material for: miR-629-3p may serve as a novel biomarker and potential therapeutic target for lung metastases of triple-negative breast cancer
Source: Breast Cancer Res. 2017 Jun 19;19:72. doi: 10.1186/s13058-017-0865-y (PMC5477310; doi:10.1186/s13058-017-0865-y)
Supplement: Supplementary file 2 — Sequences and structures of anti-miR-629-3p lentiviral vectors, pre-miR-629 lentiviral vectors, and their corresponding scrambled vectors. (PDF 631 kb) [file 13058_2017_865_MOESM2_ESM.pdf]

anti-miR-629 vectors (Lv-anti-miR-629)

GeneCopoeia™ Expressway to Discovery
9620 Medical Center Drive, Suite 101
Rockville, MD 20850
Tel: 301-762-0888
Fax: 301-762-3888
Web: www.genecopoeia.com
Email: sales@genecopoeia.com

miArrest™ miRNA inhibitor Expression Clone Datasheet

Clone Information

Catalog No.: HmiR-AN0738-AM03
Mature miRNA Name: hsa-miR-629-3p
Mature miRNA Accession No.: MIMAT0003298
Mature miRNA Sequence: GUUCUCCCAACGUAAGCCCAGC

Vector: pEZX-AM03 (HIV based)
Antibiotic: Ampicillin
Reporter Gene: mCherry Fluorescent Protein
Whole Plasmid Size: 8938 bp
Stable Selection Marker : Hygromycin
Promoter : H1

Vector Information

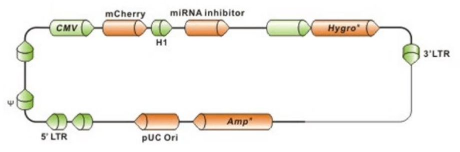

Representative structure of miArrest™ miRNA inhibitor

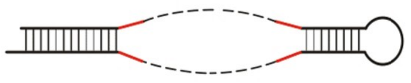

Scramble 1 vector (Lv-Scr 1)

GeneCopoeia™ Expressway to Discovery
9620 Medical Center Drive, Suite 101
Rockville, MD 20850
Tel: 301-762-0888
Fax: 301-762-3888
Web: www.genecopoeia.com
Email: sales@genecopoeia.com

miArrest™ miRNA inhibitor Expression Clone Datasheet

Clone Information

Catalog No.: CmiR-AN0001-AM03
Description: miRNA inhibitor control

Vector: pEZX-AM03 (HIV based)
Antibiotic: Ampicillin
Reporter Gene: mCherry Fluorescent Protein
Cloning Site at 5': BamHI
Whole Plasmid Size: 8936 bp
Stable Selection Marker : Hygromycin
Promoter : H1
Cloning Site at 3': EcoRI

Vector Information

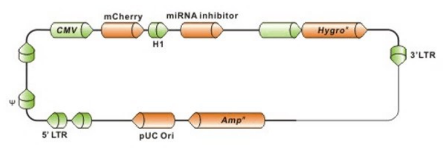

Representative structure of miArrest™ miRNA inhibitor

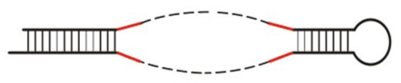

General Information:

pre-miR-629 vectors (Lv-miR-629)

GeneCopoeia™ Expressway to Discovery
9620 Medical Center Drive, Suite 101
Rockville, MD 20850
Tel: 301-762-0888
Fax: 301-762-3888
Web: www.genecopoeia.com
Email: sales@genecopoeia.com

miExpress™ Precursor miRNA Expression Clone Datasheet

Clone Information

Catalog No.: HmiR0256-MR03
Precursor miRNA Accession : MI0003643
Description: Homo sapiens miR-629 stem-loop
Vector: pEZX-MR03 (HIV based)
Antibiotic: Ampicillin
Reporter Gene: eGFP
Precursor miRNA Name: hsa-mir-629
Whole Plasmid Size: 8142 bp
Stable Selection Marker : Puromycin
Promoter : CMV

Precursor Sequence:
ucccuuucccaggggagggcugguuuacguugggagaacuuuuacggugaaccaggagguucucccaacguaagccca
gccccuccccucugccu
Mature miRNA ID: hsa-miR-629-5p
Mature miRNA Accession: MIMAT0004810
Mature miRNA Sequence: ugguuuacguugggagaacu
Mature miRNA ID: hsa-miR-629-3p
Mature miRNA Accession: MIMAT0003298
Mature miRNA Sequence: guucucccaacguaagcccagc

Suggested Sequencing Primers
Forward: 5'-CCGACAACCACTACCTGA-3'
Reverse: 5'-ATTGTGGATGAATACTGCC-3'

Vector Information for HmiR0256-MR03

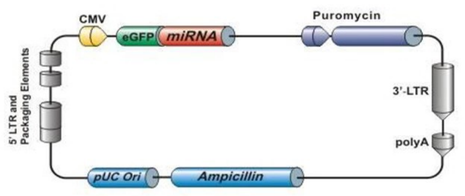

Scramble 2 vector (Lv-Scr 2)

GeneCopoeia™ Expressway to Discovery
9620 Medical Center Drive, Suite 101
Rockville, MD 20850
Tel: 301-762-0888
Fax: 301-762-3888
Web: www.genecopoeia.com
Email: sales@genecopoeia.com

OmicsLink™ miRNA Expression Clone Datasheet

Clone Information

Catalog No.: CmiR0001-MR03
Description: miRNA control
Vector: pEZX-MR03 (HIV based)
Antibiotic: Ampicillin
Reporter Gene: eGFP
Whole Plasmid Size: 8284 bp
Stable Selection Marker : Puromycin
Promoter : CMV

Suggested Sequencing Primers
Forward: 5'-CCGACAACCACTACCTGA-3'
Reverse: 5'-CGTGAAGAATGTGCGAGAC-3'

Vector Information for CmiR0001-MR03

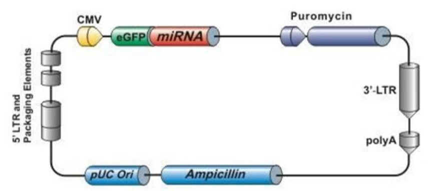

Restriction Enzyme Information for CmiR0001-MR03

Table 1. Restriction Enzymes That Do Not Cleave CmiR0001-MR03

Bsu36I<br>PmlI<br>XmaI	FseI<br>PshAI	HpaI<br>PstI	MluI<br>SbfI	NdeI<br>SgrAI	NheI<br>SmaI	PacI<br>SrfI	PflMI<br>SwaI
