# Supplementary material for: miR-629-3p may serve as a novel biomarker and potential therapeutic target for lung metastases of triple-negative breast cancer
Source: Breast Cancer Res. 2017 Jun 19;19:72. doi: 10.1186/s13058-017-0865-y (PMC5477310; doi:10.1186/s13058-017-0865-y)
Supplement: Supplementary file 3 — Characteristics of patients with TNBC in the training set and validation set. (PDF 142 kb) [file 13058_2017_865_MOESM3_ESM.pdf]

Table S1 Characteristics of patients with TNBC in training set and validation set

Variables	Training Set			Validation Set		*P*
	Lung-metastasis (n=68)	Recurrence-free (n=68)	%	n=525	%	
Age (years)						0.228
<35	6	12	13.2	72	13.7	
35-65	60	53	83.1	412	78.5	
>65	2	3	3.7	41	7.8	
Menopause						0.517
No	39	47	63.2	316	60.2	
Yes	29	21	36.8	209	39.8	
T						0.537
T1	15	28	31.6	187	35.6	
T2	36	35	52.2	262	49.9	
T3	14	3	12.5	49	9.3	
T4	3	2	3.7	27	5.1	
N						0.120
N0	8	40	35.3	238	45.3	
N1	21	18	28.7	146	27.8	
N2	17	6	16.9	70	13.3	
N3	22	4	19.1	71	13.5	
TNM stage						0.194
I	5	23	20.6	123	23.4	
II	20	33	39.0	233	44.4	
III	43	12	40.4	169	32.2	
Surgery						0.603
BCS	11	14	18.4	107	20.4	
Mastectomy	57	54	81.6	418	79.6	
Chemotherapy						0.093
No	5	3	5.9	56	10.7	
Yes	63	65	94.1	469	89.3	
Grade						0.277
I	7	10	12.5	85	16.2	
II	24	25	36.0	208	39.6	
III	37	33	51.5	232	44.2	
Ki-67						0.999
≤14%	10	26	26.5	139	26.5	
>14%	58	42	73.5	386	73.5	
LVI						0.258
Negative	43	64	78.7	435	82.9	
Positive	25	4	21.3	90	17.1	
