# Supplementary figures and images for: miR-629-3p may serve as a novel biomarker and potential therapeutic target for lung metastases of triple-negative breast cancer
Source: Breast Cancer Res. 2017 Jun 19;19:72. doi: 10.1186/s13058-017-0865-y (PMC5477310; doi:10.1186/s13058-017-0865-y)

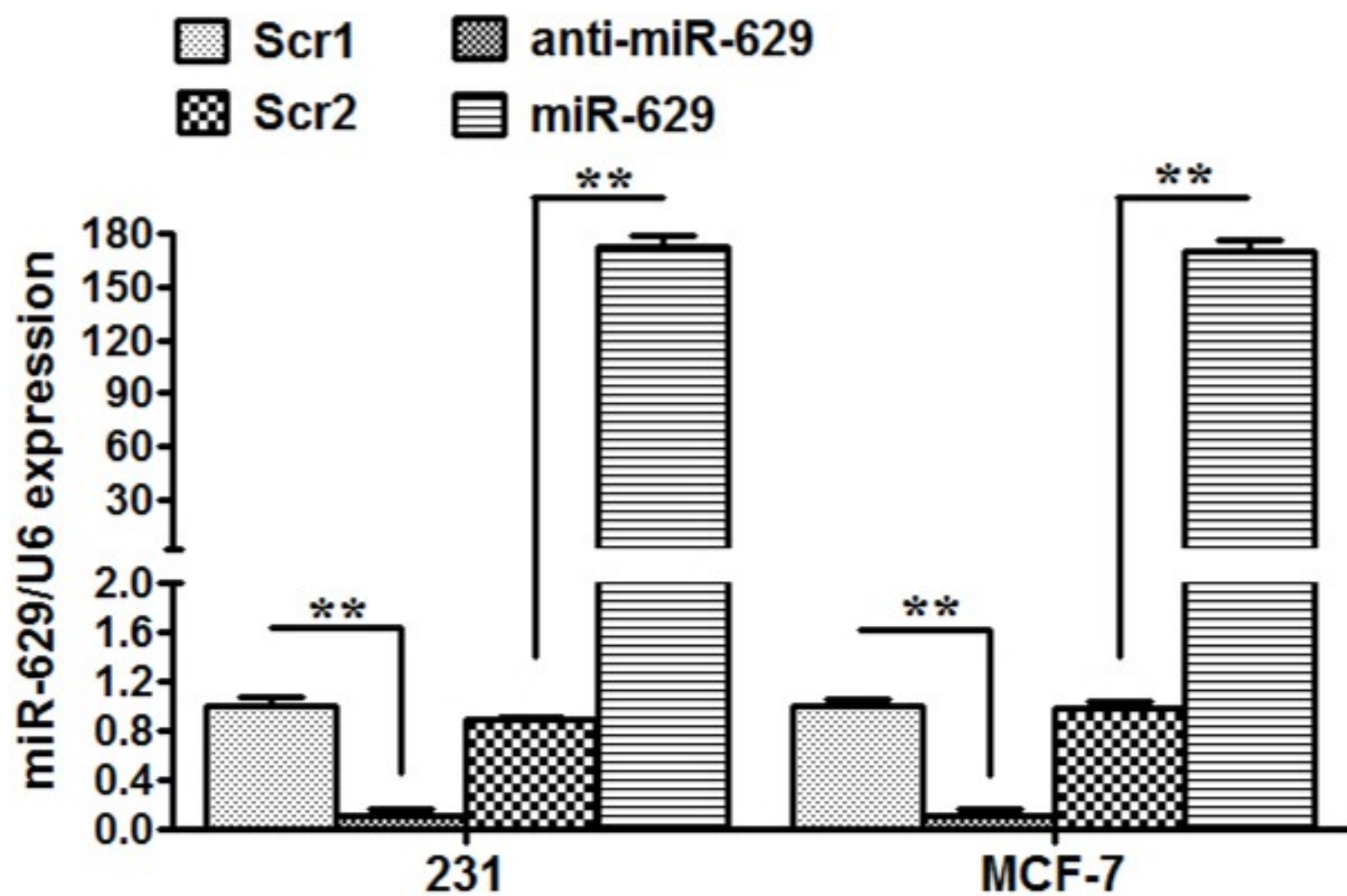

Supplement: Supplementary file 7 — Transfection of miR-629, anti-miR-629, Scr1, and Scr2 in MDA-MB-231 and MCF-7 cells. (PDF 53 kb) [file 13058_2017_865_MOESM7_ESM.pdf]
